# The Evolutionary History of the *Arabidopsis arenosa* Complex: Diverse Tetraploids Mask the Western Carpathian Center of Species and Genetic Diversity

**DOI:** 10.1371/journal.pone.0042691

**Published:** 2012-08-03

**Authors:** Roswitha Schmickl, Juraj Paule, Johannes Klein, Karol Marhold, Marcus A. Koch

**Affiliations:** 1 Department of Biodiversity and Plant Systematics, Centre for Organismal Studies (COS) Heidelberg, University of Heidelberg, Heidelberg, Germany; 2 Senckenberg Research Institute, Frankfurt am Main, Germany; 3 Institut für Spezielle Botanik und Botanischer Garten, Johannes Gutenberg University of Mainz, Mainz, Germany; 4 Department of Vascular Plant Taxonomy, Institute of Botany SAS, Bratislava, Slovakia; Montreal Botanical Garden, Canada

## Abstract

The *Arabidopsis arenosa* complex is closely related to the model plant *Arabidopsis thaliana*. Species and subspecies in the complex are mainly biennial, predominantly outcrossing, herbaceous, and with a distribution range covering most parts of latitudes and the eastern reaches of Europe. In this study we present the first comprehensive evolutionary history of the *A. arenosa* species complex, covering its natural range, by using chromosome counts, nuclear AFLP data, and a maternally inherited marker from the chloroplast genome [*trn*L intron (*trn*L) and *trn*L/F intergenic spacer (*trn*L/F-IGS) of tRNA^Leu^ and tRNA^Phe^, respectively]. We unravel the broad-scale cytogeographic and phylogeographic patterns of diploids and tetraploids. Diploid cytotypes were exclusively found on the Balkan Peninsula and in the Carpathians while tetraploid cytotypes were found throughout the remaining distribution range of the *A. arenosa* complex. Three centers of genetic diversity were identified: the Balkan Peninsula, the Carpathians, and the unglaciated Eastern and Southeastern Alps. All three could have served as long-term refugia during Pleistocene climate oscillations. We hypothesize that the Western Carpathians were and still are the cradle of speciation within the *A. arenosa* complex due to the high species number and genetic diversity and the concurrence of both cytotypes there.

## Introduction

Pleistocene climatic fluctuations strongly shape the evolutionary history of new species (e.g., grasshoppers [1]) and the distribution of genetic variants at the population level within species (e.g., *Arabidopsis* [2] and hominins [3]). In numerous studies of the European flora and fauna, these aspects of Pleistocene climatic oscillations have been investigated, but mainly confined to a single species. In this study we consider a species complex of wild relatives of the model plant *Arabidopsis thaliana* (L.) Heynh. with various taxa and cytotypes to investigate the hypothesis that past and ongoing gene flow between taxa and ploidal levels contributes to the ability of populations to adapt and survive in rapidly changing environments, particularly during the Pleistocene [4], [5].

The *Arabidopsis arenosa* species complex is one of three major species complexes within the genus *Arabidopsis* [6]–[10], formerly treated as *Cardaminopsis* [6], [7]. *Arabidopsis arenosa* and its segregates might not only represent the most ancestral species complex compared to the other two major species lineages, namely *Arabidopsis lyrata* (L.) O'Kane & Al-Shehbaz and *A. halleri* (L.) O'Kane & Al-Shehbaz [9], but the various taxa also harbor greater genetic diversity than any other *Arabidopsis* species and show a remarkable broad spectrum of ecological adaptations from high alpine regions in the High Tatras to sand dune vegetation in Scandinavia [9], [10]. A taxonomic overview of the various taxa of the *A. arenosa* species complex, including ploidal level and geographic distribution, is provided in Table 1. *Arabidopsis arenosa* is a colline, montane, and subalpine species complex with a mainly Central European distribution range including parts of the Alps and Carpathians. Only a few studies have been attempted to unravel the evolutionary history of the *A. arenosa* complex [9], [10]. Several studies focused on the natural hybrid *A. suecica* (Fr.) Norrl., which is of allopolyploid origin with the maternal parent *A. thaliana* and a paternal parent from the *A. arenosa* species complex [11], [12], confirmed also by artificial crosses [13]. Polyploidisation, mainly tetraploidisation, is frequent in several taxa of the *A. arenosa* complex [14], indicating repeated independent polyploidisation events. Introgression, the stable integration of genetic material from one species into another through repeated backcrossing, was observed between members of the *A. arenosa* and *A. lyrata* complexes [5], [15].

**Table 1:** Taxonomy, ploidal level, and geographic distribution of the various taxa of the *Arabidopsis arenosa* species complex (for details refer to the text).

Taxon	Ploidal level	Distribution range
*Arabidopsis arenosa* (L.) Lawalrée		
subsp. *arenosa*	2*n* = 16/32	Central and Western Europe, Scandinavia (lower altitudes)
subsp. *arenosa* var. *intermedia* (Kovats) Hayek	2*n* = 32	Southeastern Austrian Alps
subsp. *borbasii* (Zapałowicz) O'Kane & Al-Shehbaz	2*n* = 32	Central and Western Europe (mountain ranges, higher altitudes)
*Arabidopsis carpatica*, nom. prov.	2*n* = 16	Carpathians (limestone)
*Arabidopsis croatica* (Schott) O'Kane & Al-Shehbaz	2*n* = 16/32	Bosnia, Croatia
*Arabidopsis neglecta* (Schultes) O'Kane & Al-Shehbaz		
subsp. *neglecta*	2*n* = 16	Carpathians (alpine ranges)
subsp. *robusta*, nom. prov.	2*n* = 32	Carpathians (alpine ranges, only occasionally in lower altitudes)
*Arabidopsis nitida*, nom. prov.	2*n* = 16	Carpathians (mountain ranges, middle to subalpine altitudes)
*Arabidopsis petrogena* (A. Kern) V.I. Dorof.		
subsp. *petrogena*	2*n* = 16	Carpathians
subsp. *exoleta*, nom. prov.	2*n* = 32	Carpathians

Several taxa are awaiting taxonomic recognition (indicated with nom. prov.).

According to different authors, the *Arabidopsis arenosa* complex comprises several taxa at various taxonomic levels. The complex has been treated as one species [*A. arenosa* (L.) Lawalrée] with two subspecies of partly overlapping distribution ranges in Central Europe [16]: the mainly tetraploid subsp. *arenosa* (2*n* = 16/32), also occurring in northern Europe, growing mainly on siliceous bedrock and sandy soil, and the tetraploid subsp. *borbasii* (Zapał.) O'Kane & Al-Shehbaz (2*n* = 32), growing predominantly on calcareous bedrock and additionally found in the Carpathians. Diploid *A. neglecta* (Schult.) O'Kane & Al-Shehbaz (2*n* = 16) was described mainly from the Carpathians and rarely from the Alps, but its occurrence in the Alps is doubtful, since in the Alps this taxon has been introduced as *Cardaminopsis arenosa* var. *intermedia* (Kovats) Hayek [17]. Based on morphological and karyological data, several additional, mainly diploid Carpathian taxa at the species and subspecies level have been proposed, which were at that time attributed to the genus *Cardaminopsis* [14], [18]. These names were, however, never validly published and kept as nomina provisoria (nom. prov.) [19], pending ongoing studies aimed at clarifying their exact taxonomic status: *Arabidopsis carpatica*, nom. prov. (2*n* = 16), *A. nitida*, nom. prov. (2*n* = 16), *A. petrogena* (A. Kern) V.I. Dorof. subsp. *petrogena* (2*n* = 16), and *A. petrogena* subsp. *exoleta*, nom. prov. (2*n* = 32). In general, taxonomic concepts in the *A. arenosa* species complex are strongly debated [10].

There is an increasing interest in *A. arenosa* as a model system for adaptation to calcareous versus siliceous bedrocks (Koch and Widmer, ongoing studies; Bomblies et al., ongoing studies), or character trait research such as shade-tolerance (Bomblies et al., ongoing studies). Additionally, *A. arenosa* is interesting in terms of hybrid speciation, as it is the paternal parent of the natural allopolyploid *A. suecica*. Studies on the genomic consequences of hybridization are underway, and a first assembly of the *A. arenosa* genome is available (wiki.bioinformatics.ucdavis.edu/index.php/Arabidopsis_arenosa_whole_genome_assembly). Working with *A. arenosa* as a model system needs careful consideration of the evolutionary history of the taxa one is investigating, particularly the distribution of natural genetic variation within and among taxa.

The following two aspects are the focus of our research: Unravelling the broad-scale cytogeographic and phylogeographic patterns of diploids and tetraploids: We are particularly interested in contact zones of populations with different or mixed ploidal levels, as they can indicate ongoing species differentiation. The second is detecting centers of genetic diversity: In the northern hemisphere late Quaternary climate oscillations, especially the last glacial maximum (LGM), about 26,500 to 19,000–20,000 years ago, had the most severe influence on present-day distribution and diversity of plant taxa. *Arabidopsis arenosa* is distributed both in regions that remained largely unglaciated during Pleistocene climate oscillations and in areas formerly covered by glaciers, making it well-suited for comparative studies of evolution in changing environments.

## Materials and Methods

### Plant material

**Figure 1:**
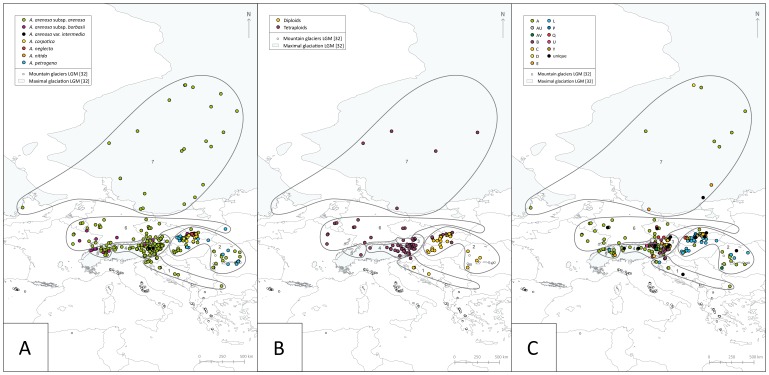
Distribution of accessions from the *Arabidopsis arenosa* species complex investigated. Maximal glaciation and mountain glaciers of the LGM are drawn according to Ehlers and Gibbard [32]. The borders of the seven geographic regions are indicated (see also Table S1, where the affiliation of each accession to one of these regions is listed). A: Visualization according to taxonomy. Seven entities are distinguished: *A. arenosa* subsp. *arenosa*, *A. arenosa* subsp. *borbasii*, *A. carpatica*, *A. neglecta*, *A. nitida*, and *A. petrogena*, following Měsíček [14], [18] and Kolník [19], and *Arabidopsis arenosa* var. *intermedia* from the Alps. B: Visualization according to ploidal level (diploids and tetraploids). Ploidal level estimates were only available for a subset of accessions. C: Visualization according to chloroplast DNA suprahaplotypes.

The accession list is provided in Table S1. Geographic distribution of single accessions is shown in Fig. 1A. Ploidy information was obtained from 214 accessions (126 populations, one to eleven individuals with five flowers each), AFLP data from 356 accessions (275 populations, one to seven individuals each), and plastid *trn*L/F sequence data from 365 accessions (260 populations, one to eleven individuals each) (Table S1).

### Mitotic chromosome preparations

Ploidy was determined both in this and a previous publication [5] and additionally from herbarium vouchers from the Herbarium of the Natural History Museum Vienna on which Polatschek had indicated chromosome numbers. The respective source of ploidy determination is recorded in Table S1. Cytological methods and light microscopy were applied according to Schmickl et al. [5].

### DNA isolation and amplified fragment length polymorphisms (AFLPs)

Total DNA was obtained from dried leaf material and extracted according to a CTAB protocol [20] with modifications according to previous studies [4].

AFLP analysis was performed using a standard protocol [21] with the following modifications: Approximately 200–500 ng DNA was digested and ligated in a 15 µl reaction mix containing T4 ligase buffer and ATP solution (Bioline, USA), 50 mM NaCl, 0.75 µg BSA, 1.5 U T4 ligase (Bioline, USA), 1 U MseI and 5 U EcoRI (New England Biolabs, USA), 0.37 µM EcoRI adapter and 3.67 µM MseI adapter. The reaction mix was incubated for 3 h at 37°C, followed by an inactivation step for 10 minutes at 65°C. The restriction-ligation product was subsequently diluted tenfold. In the pre-selective PCR 2.5 µl of the diluted restriction-ligation product was used in a total reaction volume of 12.5 µl containing PCR buffer II [Applied Biosystems (ABI), USA], 2 mM MgCl_2_, 0.8 mM dNTP mix, 0.2 µM EcoRI-A primer (5′-GAC TGC GTA CCA ATT CA-A-3′), 0.2 µM MseI-C primer (5′-GAT GAG TCC TGA GTA AC-C-3′), and 0.25 U AmpliTaq polymerase (ABI). The reactions were held at 72°C for 2 min followed by 20 cycles of 94°C for 20 s, 56°C for 30 s, and 72°C for 2 min with a final 30 s extension at 60°C. The pre-selective PCR product was visualized on a 1.5% agarose gel and diluted tenfold. For selective PCR we used 2.5 µl of the diluted pre-selective PCR product as template in a total reaction volume of 12.5 µl. The PCR mix contained 1× GoldTaq buffer (ABI), 2.5 mM MgCl_2_, 0.8 mM dNTP mix, 0.08 µM EcoRI fluorescence labelled primer, 0.2 µM Mse primer [EcoRI-AGG(TET)/MseI-CTC, EcoRI-AAC(6-FAM)/MseI-CTG, EcoRI-AAG(HEX)/MseI-CAC], and 0.5 U AmpliTaq Gold (ABI). The reactions were held at 95°C for 5 min followed by 13 cycles of 94°C for 30 s, 65°C→56°C (−0.7°C per cycle) for 1 min, and 72°C for 1 min, followed by 23 cycles of 94°C for 30 s, 56°C for 1 min, and 72°C for 1.5 min with a final 8 min extension at 72°C.

Three differentially fluorescence labelled PCR products of the same sample were multiplexed and diluted, and the fragments were electrophoretically separated on a MegaBase 500 sequencer together with an ET-ROX 550 size standard (Amersham Biosciences, USA). For each run a total of 48 samples were analyzed, including one standard sample, one negative control, one repeat within the runs, and several other replicates (altogether 6.5%), as recommended by Bonin et al. [22]. Raw data were visualized and the fragments in the range of 60–513 bp manually scored using GeneMarker version 1.9 (SoftGenetics, USA). Processed data were exported as a presence/absence matrix.

### *Trn*L/F amplification and sequencing

For the cpDNA markers *trn*L intron and *trn*L/F intergenic spacer (*trn*L/F-IGS), primers, PCR cycling scheme, purification of the amplified fragment, cycle sequencing, and sequencing on a MegaBace 500 sequencer followed the protocol of Schmickl et al. [4]. Amplified sequences of *trn*L/F-IGS included the complete *trn*L/F-IGS and the first 18 bases of the *trn*F gene.

### AFLP genetic diversity statistics and Principal Component Analysis (PCA)

Several statistical parameters were computed using the R script AFLPdat [23], R 2.9.2 environment [24] for geographic and taxonomic groups: proportion of variable markers (FP) and Nei's gene diversity (*H_E_* [25]). The following seven geographic regions were considered: (1) Balkan Peninsula (Balk), (2) Carpathians (Carp), (3) unglaciated Eastern and Southeastern Alps (UnglaESEAlps), (4) glaciated Eastern Alps (GlaEAlps), (5) glaciated Western Alps (GlaWAlps), (6) unglaciated Central Europe (UnglaCentrEur), and (7) glaciated northern Europe (GlaNEur). These regions are illustrated in Figure 1. Regarding taxonomy, six entities were distinguished following Měsíček [14], [18] and Kolník [19]: *A. arenosa* subsp. *arenosa*, *A. arenosa* subsp. *borbasii*, *A. carpatica*, *A. neglecta*, *A nitida*, and *A. petrogena*. In order to analyze and display the similarity among the AFLP genotypes, a Principal Component Analysis (PCA) was performed using MVSP version 3.1 (Kovach Computing Services, UK). Pairwise Euclidean distance was applied as distance measure and, alternatively, both Jaccard and simple match coefficient [26].

### Plastid *trn*L/F sequence definition, network analysis, genetic diversity statistics, and map reconstruction

Plastid *trn*L/F sequences were defined as i) haplotypes and ii) suprahaplotypes following our previous studies (e.g., [4]). Haplotypes (i) are characterized by varying (in sequence and structure) *trn*F pseudogenes in the 3′-region of the *trn*L/F-IGS close to the functional *trn*F gene: Haplotypes belonging to one suprahaplotype (ii) share the same base order throughout the whole sequence except for the pseudogene-rich region, where they vary in both length and base content. Mutation rate within the pseudogene-rich region is about 10 to 20 times higher than within the non-coding spacer and intron regions [27]. Therefore, our cpDNA dataset is based on *trn*L/F suprahaplotypes only. Suprahaplotypes differ from each other by single point mutations and/or indels. Newly defined *trn*L/F haplotypes were assigned to GenBank numbers [FJ477684–FJ477690, FJ477705–FJ477716] (Table S1). The network was constructed using TCS version 1.21 [28] using the statistical parsimony algorithm [29]. Single gaps (except polyT stretches) were coded as single additional binary characters. Genetic diversity statistics were performed with Arlequin version 3.11 [30]: Genetic diversity was estimated as effective genetic diversity according to Gregorius (*V_a_*) [31], nucleotide diversity *π* and Nei's unbiased gene diversity *H_E_*.

In order to visualize the geographical data, ArcView version 8.2 (ESRI, USA) was used. The maximum extent of the ice sheets during the LGM was taken from Ehlers and Gibbard [32].

## Results

### Chromosome counts identify diploids exclusive to the Balkan Peninsula and the Carpathian Mountains

Two ploidal levels, diploid and tetraploid, were observed within our sampling (Fig. 1B). Diploids were exclusively found in southeastern and eastern Europe on the Balkan Peninsula, in northern Hungary, and in the Carpathians. In contrast, tetraploids have a large distribution range and occur from the Julian Alps (Slovenia) in the south, the Western Carpathians (Slovakia) in the east, France and Belgium in the west, and Scandinavia in the north. Several regions were reported as areas of recent, mainly anthropogenically influenced colonisation after 1890 [33] (Belgium, Finland, France, Great Britain, Greenland), frequently along railway tracks. The Balkan Peninsula and the Western Carpathians were the only regions where both diploid and tetraploid populations were found. Populations of mixed ploidal levels were not observed, but can not be completely ruled out, as only a limited number of populations from the Eastern Alps (*n* = 28) and the Western Carpathians (*n* = 9) were analyzed with more than one individual per population.

### AFLP data indicate similar values for gene diversity throughout Europe and demonstrate high gene diversity of a widespread tetraploid

Diversity statistics, based on AFLP data, showed similar values for gene diversity throughout the whole distribution range of the *A. arenosa* species complex (Table 2), ranging from *H_E_* = 0.133 (GlaNEur) to *H_E_* = 0.159 (UnglaESEAlps). The proportion of variable markers differed more strongly between geographic regions, ranging from FP = 0.411 (Balk) to FP = 0.906 (UnglaESEAlps). However, the proportion of variable markers is biased with respect to sample size (Balk: *n* = 9, UnglaESEAlps: *n* = 114) and, therefore, not a valid measurement of genetic diversity. Genetic diversity patterns in the *A. arenosa* complex need additional consideration in terms of taxonomy, as for certain regions, e.g., Carp, numerous taxa are described, and in other regions, e.g., GlaNEur, only one taxon is found. Gene diversity of the different taxa ranged from *H_E_* = 0.157 (*A. arenosa* subsp. *arenosa*) and *H_E_* = 0.155 (*A. arenosa* subsp. *borbasii*) to *H_E_* = 0.138 (*A. carpatica* and *A. petrogena*) and *H_E_* = 0.125 (*A. neglecta*). The proportion of variable markers is, again, highly correlated with sample size (*A. neglecta*: *n* = 6, FP = 0.296; *A. arenosa* subsp. *arenosa*: *n* = 277, FP = 0.985) and so should be treated with caution.

**Table 2:** (a) Regional genetic differentiation and (b) genetic differentiation according to taxonomy, based on AFLP and chloroplast DNA sequence data (*trn*L/F suprahaplotypes).

(a) Geographic region	AFLPs	AFLPs	AFLPs	*trn*L/F	*trn*L/F	*trn*L/F	*trn*L/F
	*n*	Nei's gene diversity (*H_E_*)	Proportion of variable markers (FP)	*n*	*V_a_*	Nucleotide diversity (*π*×10^−2^)	Nei's gene diversity (*H_E_*)
Balk	9	0.144	0.411	10	2.94	0.395+/−0.254	0.733+/−0.101
Carp	88	0.144	0.885	107	3.49	0.352+/−0.208	0.719+/−0.030
UnglaESEAlps	114	0.159	0.906	132	4.14	0.483+/−0.271	0.764+/−0.024
GlaEAlps	50	0.143	0.740	51	2.23	0.310+/−0.190	0.561+/−0.076
GlaWAlps	32	0.144	0.661	19	1.24	0.109+/−0.090	0.205+/−0.119
UnglaCentrEur	46	0.151	0.773	35	1.64	0.241+/−0.157	0.403+/−0.102
GlaNEur	17	0.133	0.480	11	1.81	0.315+/−0.209	0.491+/−0.175

(b) Taxon	AFLPs	AFLPs	AFLPs	*trn*L/F	*trn*L/F	*trn*L/F	*trn*L/F
	*n*	Nei's gene diversity (He)	Proportion of variable markers (FP)	*n*	*V_a_*	Nucleotide diversity (*π*×10^−2^)	Nei's genetic diversity (*H_E_*)
*A. arenosa* subsp. *arenosa*	277	0.157	0.985	282	3.48	0.410+/−0.235	0.714+/−0.023
*A. arenosa* subsp. *borbasii*	20	0.155	0.622	14	1.34	0.111+/−0.093	0.275+/−0.148
*A. carpatica*, nom. prov.	29	0.138	0.651	37	3.71	0.514+/−0.293	0.751+/−0.054
*A. neglecta*	6	0.125	0.296	6	2.00	0.470+/−0.319	0.600+/−0.215
*A. petrogena*	23	0.138	0.602	24	2.42	0.241+/−0.159	0.612+/−0.089

Sample size (*n*), Nei's gene diversity (*H_E_*), proportion of variable markers (FP), and nucleotide diversity (*π*) with standard deviation are provided. For *trn*L/F suprahaplotypes effective genetic diversity according to Gregorius (*V_a_*) is additionally displayed. The following seven geographic regions were considered: (1) Balkan Peninsula (Balk), (2) Carpathians (Carp), (3) unglaciated Eastern and Southeastern Alps (UnglaESEAlps), (4) glaciated Eastern Alps (GlaEAlps), (5) glaciated Western Alps (GlaWAlps), (6) unglaciated Central Europe (UnglaCentrEur), and (7) glaciated northern Europe (GlaNEur). *Arabidopsis arenosa* var. *intermedia* is integrated within *A. arenosa* subsp. *arenosa*. *Arabidopsis nitida* was omitted from the analyses, as it was represented by one (AFLPs) and three (*trn*L/F suprahaplotypes) accession(s) only.

**Figure 2:**
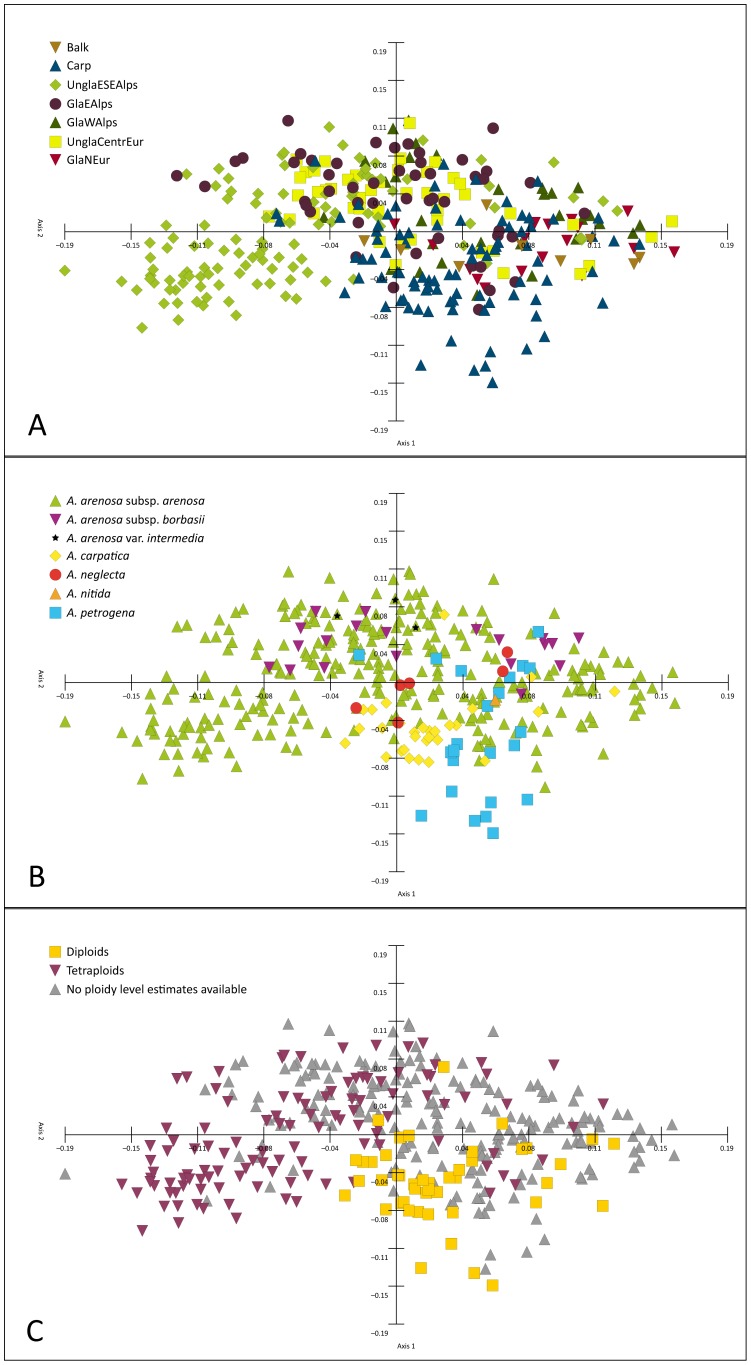
Principal Component Analysis of AFLP data from the *Arabidopsis arenosa* species complex. Each symbol represents an individual. A: Visualization according to geographic regions. The following seven geographic regions were considered: (1) Balkan Peninsula (Balk), (2) Carpathians (Carp), (3) unglaciated Eastern and Southeastern Alps (UnglaESEAlps), (4) glaciated Eastern Alps (GlaEAlps), (5) glaciated Western Alps (GlaWAlps), (6) unglaciated Central Europe (UnglaCentrEur), and (7) glaciated northern Europe (GlaNEur). These regions are illustrated in Figure 1. B: Visualization according to taxonomy. *Arabidopsis arenosa* var. *intermedia* is marked with an asterisk. C: Visualization according to ploidal level. Data lacking ploidal level estimates are marked in grey.

PCA according to regions (Fig. 2A) resulted in overlapping groups of AFLP genotypes from nearly all regions, except for accessions from parts of Carp and UnglaESEAlps. Groups of AFLP genotypes according to taxonomy also largely overlapped (Fig. 2B). Widespread *A. arenosa* subsp. *arenosa* but also subsp. *borbasii* formed large groups of AFLP genotypes in comparison to *A. carpatica*, *A. neglecta*, and *A. petrogena* (*A. nitida* was omitted, as it was represented by one accession only). This finding underlines the high genetic plasticity of the tetraploids (*A. arenosa* subsp. *arenosa* and subsp. *borbasii*) in contrast to the mainly diploids [*A. carpatica* (exclusively diploid), *A. neglecta* (predominantly diploid), *A. petrogena* (partially diploid)]. PCA according to ploidal levels (Fig. 2C) revealed two partly overlapping clusters of diploids and tetraploids, but due to many accessions without ploidal level estimates the two clusters could actually be more strongly intermingled.

### The Balkan Peninsula, the Carpathians and the unglaciated Eastern and Southeastern Alps are the three centers of chloroplast sequence diversity of the *A. arenosa* species complex

Based on *trn*L/F sequence data, we detected three centers of genetic diversity of the *A. arenosa* complex (Table 2). The unglaciated Eastern and Southeastern Alps displayed highest effective diversity according to Gregorius (*V_a_* = 4.14), highest nucleotide diversity (*π* = 0.483%), and highest Nei's gene diversity (*H_E_* = 0.764) within the whole dataset. The Balkan Peninsula was detected as the second center of genetic diversity (*V_a_* = 2.94, *π* = 0.395%, *H_E_* = 0.733). The third center of genetic diversity, the Carpathians, was characterized by gene diversity values similar to those of the Balkan Peninsula (*V_a_* = 3.49, *π* = 0.352%, *H_E_* = 0.719). In contrast to these three genetically highly diverse regions, which remained largely unglaciated during Pleistocene climate oscillations, formerly glaciated regions showed reduced values of effective genetic diversity according to Gregorius, nucleotide and Nei's gene diversity: The part of the Eastern Alps formerly covered by glaciers was characterized by *V_a_* = 2.23, *π* = 0.310%, and *H_E_* = 0.561. The formerly glaciated Western Alps showed strongly reduced values (*V_a_* = 1.24, *π* = 0.109%, *H_E_* = 0.205). Glaciated northern Europe was characterized by *V_a_* = 1.81, *π* = 0.315%, and *H_E_* = 0.491. Although Central Europe remained largely unglaciated during Pleistocene climate oscillations, genetic diversity was also reduced (*V_a_* = 1.64, *π* = 0.241%, *H_E_* = 0.403). In contrast to AFLP data, effective genetic diversity according to Gregorius, nucleotide and Nei's gene diversity of diploid *A. carpatica* (*V_a_* = 3.71, *π* = 0.514%, *H_E_* = 0.751) exceeded that of tetraploid *A. arenosa* subsp. *arenosa* (*V_a_* = 3.48, *π* = 0.410%, *H_E_* = 0.714).

**Figure 3:**
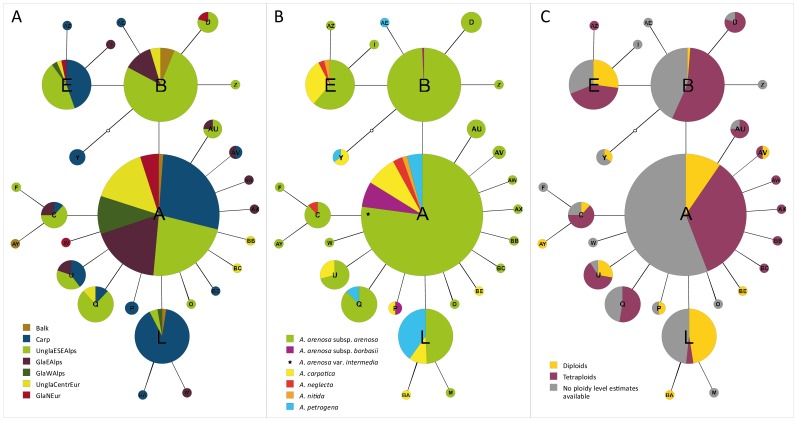
Chloroplast DNA *trn*L/F suprahaplotype networks of the *Arabidopsis arenosa* species complex. The sizes of the circles indicate the relative frequency of a suprahaplotype. Geographic regions, taxonomic entities, and cytotypes are indicated with the same colours as in Figure 2. A: Visualization according to geographic regions. B: Visualization according to taxonomy. *Arabidopsis arenosa* var. *intermedia* is marked with an asterisk. C: Visualization according to ploidal level.

In all geographic regions, except GlaWAlps, unique suprahaplotypes were found, which occurred in one region only (Figs. 1C, 3A). These unique types are nearly all derived from the “core” suprahaplotypes A, B, and E: AY (Balk); AE, AZ, BA, BE, P, Y (Carp); F, O, Z (UnglaESEAlps); AW, AX, I, M (GlaEAlps); BB, BC (UnglaCentrEur); W (GlaNEur). In contrast to the suprahaplotypes, which were shared between regions (Fig. 3A) and also between taxa (Fig. 3B; A, B, C, E, L, Q, U), these regionally unique suprahaplotypes were found in exclusively one taxon, except P and Y (Fig. 3B). Regarding ploidal levels (Fig. 3C), diploids and tetraploids shared numerous suprahaplotypes (A, AV, B, C, E, L, U) but also had unique ones (diploids: AY, BA, BE, P, Y; tetraploids: AU, AW, AX, AZ, BB, BC, D, Q).

## Discussion

### The Western Carpathian Mountains are the cradle of speciation within the *A. arenosa* complex

Speciation is often accompanied by polyploidisation [34]. Within the *A. arenosa* complex two ploidal levels, diploid and tetraploid, were observed. Contact zones of these two cytotypes were localized in the northwestern part of the Balkan Peninsula and in the Western Carpathians, and, consequently, at least one independent polyploidisation event can be assumed for each of these regions. Regarding the Western Carpathians, we observed a mosaic pattern of diploids and tetraploids, which is in congruence with Měsíček [14] (see also [19]). In the contact zones of diploids and tetraploids in the Western Carpathians we found no populations with mixed ploidal levels. This finding can be discussed with respect to the origin of the tetraploids: Populations of mixed ploidal levels could either be the result of a relatively recent autopolyploidisation event or the result of secondary contact of formerly allopatric populations with different ploidal levels. The genus *Melampodium* L., in particular the white-rayed species complex with mainly diploids and tetraploids, is an example for postglacial formation of polyploids via autopolyploidisation. Intrapopulational cytotype mixture was reported, but emphasized as rare [35]. In contrast, secondary contact of formerly allopatric populations with different ploidy is the explanation for populations of mixed ploidal levels (diploids and tetraploids or tetraploids and hexaploids) in the *Knautia arvensis* agg. [36]. Therefore, we conclude, that the lack of mixed ploidy populations and the lack of uneven ploidy and aneuploidy in the *A. arenosa* complex excludes recent polyploidisation events as well as secondary contact zones. We assume ancient polyploidisation, probably dated several glacial cycles ago. Slightly different ecological adaptations of diploids and tetraploids might have favored such a distinct pattern of diploid and tetraploid populations. Diploid *A. neglecta* subsp. *neglecta*, for example, is found on siliceous substrates, mostly in high alpine habitats above the tree line, where it grows along mountain streams. The tetraploid subspecies *robusta* (previously recognized as *Cardaminopsis neglecta* subsp. *robusta* [18], but never validated by publication), corresponds to a taxon, which, although it also occurs on siliceous substrates, is found in different mountain ranges and at lower altitudes compared to typical subspecies (mostly around or below the tree line [Kolník, unpubl. data]). This is comparable to *Senecio carniolicus* Willd. with an altitudinal, ecological gradient composed of mainly diploid and hexaploid populations in the Eastern Alps [37].

Additionally, the high genetic diversity, based on plastid *trn*L/F, in the Western Carpathians is an indicator for past and also ongoing speciation within the *A. arenosa* complex. Not only the high genetic diversity but also the high number of species and subspecies underline the Western Carpathians as a cradle of *A. arenosa* speciation. Numerous proposed taxa are unique for the Carpathians: *A. neglecta* is considered to comprise two subspecies, one diploid (subsp. *neglecta*) and one tetraploid (subsp. *robusta*) [18; Měsíček, unpubl. data]. *Arabidopsis petrogena* is exclusively described from the Carpathians as diploid (subsp. *petrogena*) and as tetraploid (subsp. *exoleta* [18; Měsíček, unpubl. data]). *Arabidopsis nitida* is the third unique taxon of the Carpathians. And within broadly defined *A. borbasii* a Carpathian subspecies (subsp. *carpatica*, referred to as *A. carpatica*, nom. prov. [19], and as *C. borbasii* subsp. *carpatica* [18]) is discussed. However, taxonomy of this highly diverse species complex needs to be revised in the near future.

### Long-term evolution in two glacial refugia: the Carpathians and the unglaciated Eastern/Southeastern Alps

In numerous studies of both plant and animal species three classical LGM refugia were reported, based on the fossil record [38] and species and genetic diversity [39]: the Balkan Peninsula, the Iberian Peninsula, and the Appenin. Out of these three Pleistocene refugia, the Balkan Peninsula was emphasized as the most important refugium, especially for tree species [40], but also for upper and lower montane taxa of especially eastern European distribution [41], [42]. The Balkan Peninsula probably served as one of the major refugial areas for the *A. arenosa* complex, which will be discussed in more detail in the following section. Although the Carpathians remained largely unglaciated during Pleistocene climate oscillations, except in the south, they have been reported as a refugium for only a few plant species so far, including temperate trees [39], [43] and herbs, e.g., *Campanula alpina* Jacq. [44]. For the *A. arenosa* complex, the Western Carpathians were already described as a cradle of speciation, and they probably served as a second major refugial area. In contrast, the unglaciated eastern Austrian Alps have long been assumed as a glacial refugium. Along the eastern border of the Austrian Alps a cryptic refugium for tree species was already suggested [45], according to palynological data and supported by phylogeographic analyses [46]. Additionally, the northeastern Limestone Alps have already been described as rich in subalpine endemics [47], and this view was further supported by Tribsch and Schönswetter [48]: Based on species diversity and the number of endemics, they suggested refugia for numerous calcicolous and silicicolous plants. As *A. arenosa* is both a colline-montane and subalpine species complex, only parts of the subalpine refugia of the northeastern Limestone Alps overlapped with the *A. arenosa* refuge area of the unglaciated Eastern and Southeastern Alps. This is in congruence with the view that the eastern edge of the northeastern Limestone Alps is an area of periglacial survival for montane plant species [49], [50].

### Parallel evolution in the Eastern Alps and the Western Carpathians

According to *trn*L/F sequence data, the unglaciated Eastern/Southeastern Alps and the Carpathians formed two distinct genetic groups: the Alps characterized by suprahaplotype B and the Carpathians by suprahaplotype L. Although other suprahaplotypes, e.g., A and E, were shared between these two mountain ranges, we assume strong barriers to gene flow between the Alps and Carpathians, due to the Pannonian Basin, which constituted a lowland barrier for montane to subalpine taxa since the Holocene warming. Long-term genetic isolation between the Alps and Carpathians was also proposed for several other plant species, such as *Campanula alpina* [44]. Additional studies from *Ranunculus glacialis* L. [51] and *Rosa pendulina* L. [52] support this view.

However, an alternative explanation for genetic differentiation between *A. arenosa* populations from the Alps and Carpathians has to be considered. Based on our karyologic data, independent colonisation of both mountain ranges from the Balkan Peninsula can be hypothesized: Compared to all other regions, exclusively diploid cytotypes (except for one accession in the northern part) were found on the Balkan Peninsula. Diploid *A. arenosa* could have migrated northwards and experienced multiple polyploidisation events, especially in the Carpathians, where taxa of both diploid and tetraploid cytotype are described. The Alps were probably colonised by (a) tetraploid cytotype(s) of *A. arenosa*, which could have originated in the Julian Alps, where only tetraploids were found. The Balkan Peninsula as the ancient refuge area for the *A. arenosa* species complex is additionally supported by the occurrence of an *Arabidopsis* species endemic to the Balkan Peninsula, *Arabidopsis croatica*. This endemic is closely related to the *A. arenosa* complex, based on ITS sequence data (internal transcribed spacer region of nuclear encoded ribosomal DNA) [9], [10].

Taxa in refuge areas like the Eastern Alps and the Western Carpathians probably underwent long-term adaptational processes, which could result in adaptation to the same ecological niche in the two mountain ranges in parallel. Indeed, we found one example for parallel evolution in the Eastern Alps and the Western Carpathians: Diploid *A. neglecta* grows on siliceous bedrock along mountain streams in alpine habitats of the High Tatras (Slovakia), a similar ecological niche in the Eastern Alps (Wölzer Tauern, Styria) is occupied by a tetraploid taxon corresponding to *Cardaminopsis arenosa* var. *intermedia* (Kovats) Hayek [17]. Besides sharing similar ecological demands, the two taxa are discussed to be morphologically more similar to each other than to any other member of the *A. arenosa* complex. However, AFLP data did not confirm that the vicariant populations from the Eastern Alps and the High Tatras represent a single species (Fig. 2B). This needs further experimental confirmation based on a broader sampling of both taxa. Interestingly, two other species from the Brassicaceae co-occur in the same or neighbouring alpine regions on siliceous bedrock along mountain streams: hexaploid *Cochlearia tatrae* Borbás in the High Tatras (Slovakia) and diploid *Cochlearia excelsa* Zahlbr. ex Fritsch in the Eastern Alps (Seckauer Tauern, Styria). In parallel to *A. neglecta* and *C. arenosa* var. *intermedia*, these two *Cochlearia* species evolved independently and most likely within the last approximately 100,000 years [53], [54]. Parallel evolution of species pairs implies effective reproductive isolation between the two mountain ranges and limited multiple immigration.

### Tetraploid *A. arenosa* subsp. *arenosa* is a highly genetically diverse taxon

Based on both AFLP and chloroplast sequence data, genetic diversity was not strongly reduced in regions formerly covered by glaciers (GlaEAlps, GlaWAlps, GlaNEur) in comparison to formerly unglaciated regions (UnglaCentrEur). In all these four regions, exclusively tetraploid *A. arenosa* subsp. *arenosa* and subsp. *borbasii* were found. Local, periglacial survival could serve as one explanation for the high genetic diversity of especially *A. arenosa* subsp. *arenosa*. In Central Europe, numerous populations are restricted to relict habitats on exposed rocks in low mountain ranges, e.g., the Black Forest, the Eifel, the Elbe Sandstone Mountains, the Harz Mountains, and the Swabian Mountains, where they often co-occur with Pleistocene relict species such as *Dianthus gratianopolitanus* Vill. [Koch, unpubl. data]. A second explanation could be the lack of genetic bottlenecks and the maintenance of large effective population sizes during postglacial migration into formerly glaciated regions, probably enhanced by the plant's biennial life cycle. In a third alternative, gene flow between different taxa and/or ploidal levels could account for the high genetic diversity in especially tetraploid *A. arenosa* subsp. *arenosa*, probably before this taxon migrated from the Eastern Alps and Western Carpathians, its putative refuge areas, to Central Europe. Gene flow between different taxa of the *A. arenosa* species complex is documented by numerous hybrids, including triploid ones, reported from the Western Carpathians [19]. Gene flow between different ploidal levels was recently described for *A. lyrata* [15]: Based on an isolation with migration model analysis [55], [56], gene flow from diploids to tetraploids and vice versa was hypothesized. We assume that all three factors could have contributed to the high genetic diversity of tetraploid *A. arenosa*, which could have resulted in its ability to be a successful coloniser on various different substrate types (e.g., limestone, sandstone, granite, basalt) in various different habitat types (“natural” sites on rocks, gravel, and sand; anthropogenically influenced sites such as railway tracks and rural areas).

## Supporting Information

Table S1 Information about accession details and experimental results: taxonomic unit, taxon name on herbarium voucher, herbarium/herbarium voucher no., accession no., geographic region, latitude/longitude, locality, collector/date of collection, ploidal level/publication or other source, AFLPs, *trn*L intron type, *trn*L intron GenBank no., *trn*L/F-IGS type, *trn*L/F-IGS GenBank no., *trn*L intron+*trn*L/F-IGS type, *trn*L intron+*trn*L/F-IGS suprahaplotype. Accessions, for which AFLP data were obtained, are marked (see AFLPs).
(XLS)
